# Recovery of Polyphenols from Grape Pomace Using Polyethylene Glycol (PEG)-Grafted Silica Particles and PEG-Assisted Cosolvent Elution

**DOI:** 10.3390/molecules24122199

**Published:** 2019-06-12

**Authors:** Ayca Seker, Baran Arslan, Shulin Chen

**Affiliations:** 1 Department of Biological Systems Engineering, Washington State University, Pullman, WA 99164-6120, USA; chens@wsu.edu; 2 Gene and Linda Voiland School of Chemical Engineering and Bioengineering, Washington State University, Pullman, WA 99164-6515, USA; baran.arslan@wsu.edu

**Keywords:** grape pomace, polyphenols, purification, silica particles, polyethylene glycol (PEG), hydrogen bonding

## Abstract

Adsorption on a functionalized surface can be an effective way of purifying polyphenols from complex plant extracts. Polymeric resins that rely on hydrophobic interactions suffer from low selectivity, weak affinity towards polyphenols, and lack tunability therefore making the purification of polyphenols less efficient. In this study, a purification process for the recovery of polyphenols from grape pomace extract was successfully developed using hydrogen bonding affinity ligands grafted on silica particles and PEG-assisted elution solvents. Bare silica (SiO_2_) and polyethylene glycol (mPEG)-grafted silica microparticles with molecular weights of 2000 and 5000 were tested to determine their polyphenol binding and release characteristics. Functionalizing the surface of bare silica with mPEG ligands increased the adsorption capacity by 7.1- and 11.4-fold for mPEG-2000 and mPEG-5000 compared to bare silica particles, respectively. This was likely due to the introduction of more polyphenol binding sites with mPEG functionalization. Altering the molecular weight (MW) of mPEG grafted on silica surfaces provided tunability in the adsorption capacity. A complete recovery of polyphenols (~99.9%) from mPEG-grafted silica particles was achieved by utilizing PEG–ethanol or PEG–water cosolvent systems. Recovered polyphenols showed up to ~12-fold antioxidant activity compared to grape pomace extract. This study demonstrates that mPEG-grafted silica particles and elution of polyphenols with PEG cosolvents can potentially be used for large-scale purification of polyphenols from complex plant extracts and simplify the use of polyphenols, as PEG facilitates remarkable solvation and is an ideal medium for the final formulation of polyphenols.

## 1. Introduction

Grapes, with annual production of more than 77 million tons, is one of the largest fruit crops in the world [1]. Winemaking utilizes almost fifty percent of worldwide grape production [1]. However, large quantities of byproducts such as pulp, seeds, and skins remain unused in the process. These byproducts, so-called grape pomace, are a good and cheap source of high-quality natural polyphenolic compounds [2]. The economic implications of polyphenols are thought to be substantial because of their potential applications in cosmetic and pharmaceutical formulations in addition to the food processing industry [3]. In recent years, polyphenols have gained significant attention due to their important role in preventing obesity, coronary heart disease, colon cancer, gastrointestinal disorders, and reducing the risk of diabetes [4,5]. It was also shown that these compounds can be utilized for topical antiaging purposes [6].

Several unit operations are currently available for the isolation of polyphenols from complex plant matrices. Solid–liquid, supercritical fluid, accelerated solvent, ultrasound-assisted, microwave-assisted, subcritical water, and enzymatic-assisted extractions are among some of the extraction methods reported in the literature [7]. However, due to the cost constraints of other techniques, solid–liquid extraction is the most commonly used method for the isolation of polyphenols from grape pomace [7]. The process, however, is not selective for polyphenols and does not meet the purity demands of the pharmaceutical industry. Therefore, a purification step is essential for the recovery of polyphenols from grape pomace extract.

Polymeric resins such as polystyrene-divinyl benzene co-polymeric resins (PS-DVB) are widely used to separate phenolic compounds from crude plant extracts due to their high surface area and correlating high adsorption capacities [8]. The separation mechanism of these resins is based on hydrophobic interactions and van der Waals forces and these interactions are known to be nonspecific [9,10]. Nonpolar polymeric resins show low selectivity towards polyphenols and form weak interactions to polar compounds such as polyphenols [11,12]. These drawbacks limit the purity of polyphenols recovered from grape pomace extract using nonpolar resins if no secondary purification method is used [12,13,14]. A few attempts using resins capable of forming hydrogen bonds improved the selectivity towards polyphenols, however, they suffer from low adsorption capacity [15,16]. Polyphenols consist of several hydroxyl and carboxyl groups and the number and position of these groups vary among the polyphenol families [17]. It is likely that the orientation of the groups will determine their affinity to polar functional groups on the adsorbents. Therefore, adsorbents grafted with tunable ligands are needed for targeted separation of individual or subgroups of polyphenols [15]. However, polymeric resins cannot provide tunable surface properties and it is tedious to prepare them with the desired surface properties.

In this work, we developed a purification method for the recovery of polyphenolic compounds using silica particles grafted with polyethylene glycol (PEG) ligands. Silica is widely used in the delivery of therapeutics and chromatography due to its robust nature with well-known aqueous based synthesis protocols and modularity [18,19]. Silica particles can be functionalized with a variety of silanes to introduce hydrophobic, charged, or polar moieties to their surfaces [20,21]. Therefore, silica was chosen as a support for the target ligand used in this study. PEG was chosen as the ligand of interest due to its benign characteristics, low toxicity, and excellent biocompatibility [22] as well as its ability to form complexes with phenols and polyphenols through a more specific type of interaction: Hydrogen bonding [23,24,25]. It is hypothesized that, with the specificity of hydrogen bonding, polyphenols with a high number of hydroxyl groups can be recovered from grape pomace extract. In addition to PEG’s biocompatibility, it carries tunable properties, such as variable hydrophilicity with changes in its molecular weight (MW) [26,27]. This study aimed to tune PEG’s MW to maximize the adsorption capacity of polyphenols extracted from grape pomace onto PEG-grafted silica particles. To our knowledge, this is the first study using PEG as an affinity ligand to capture polyphenols from grape pomace extract.

The release of adsorbed polyphenols from PEG-grafted silica particles is needed for complete recovery. A green solvent swing process was also developed in this study for the recovery of polyphenols from grape pomace. The release of polyphenols was achieved by switching from aqueous ethanol (loading) to aqueous- or ethanolic-PEG cosolvent (unloading). The recovery of polyphenols from PEG-grafted silica particles was further optimized by altering the concentration of PEG in the solution. In addition, MW of PEG used in the desorption medium was tuned to selectively recover certain polyphenols from polyphenol loaded silica particles. PEG has been commonly used in the final formulation of pharmaceutical products [28,29] and it demonstrates outstanding solubility of polyphenols compared to ethanol or water [30]. It also improved the cytotoxicity of insoluble polyphenols against target cancerous cell lines [31,32]. Therefore, PEG-assisted solvent medium can simplify downstream processing of polyphenols upon isolation from grape pomace. In summary, combining solvent extraction, purification by the use of a tunable adsorbent with affinity ligands, and PEG-assisted cosolvent recovery can be an effective and environmentally benign process for obtaining highly potent polyphenols from grape pomace.

## 2. Results and Discussion

### 2.1. Characterization of Silica Particles

Surface functionalization of silica particles with mPEG ligands was confirmed by spectroscopic and gravimetric techniques. Surface area and pore volume of bare and mPEG-grafted silica particles were determined by Brunauer–Emmett–Teller (BET) analysis.

#### 2.1.1. Fourier Transform Infrared (FTIR) Analysis

**Figure 1:**
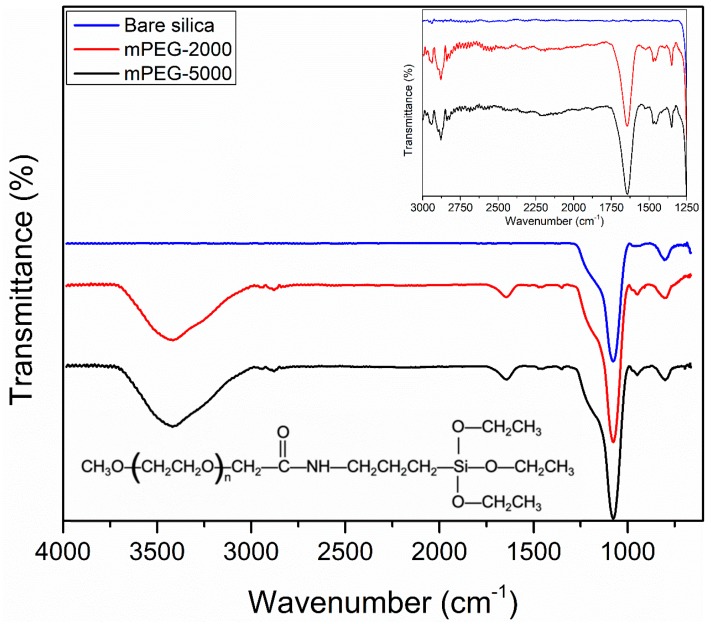
Fourier transform infrared (FTIR) spectra of bare silica and polyethylene glycol (mPEG)-grafted silica particles. Insets show the zoomed in spectra and the chemical structure of mPEG–silane.

**Figure 2:**
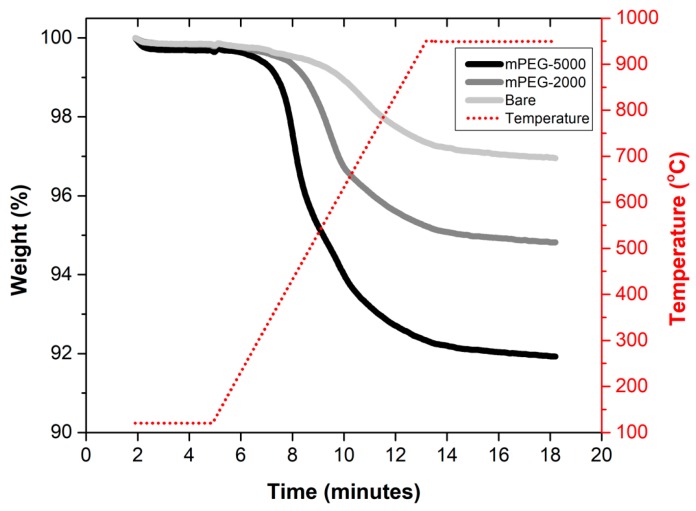
Thermogravimetric (TGA) analysis of mPEG-grafted and bare silica particles.

The surface functional groups on the surface of silica particles were confirmed based on the presence of the characteristic peaks for different moieties, such as C=O, N−H, and −CH_2_ (Figure 1). For both bare and mPEG-grafted silica particles, the peaks at ~1045 cm^−1^ are attributed to asymmetric vibrations of Si−O−Si [33]. A characteristic peak of carbonyl group (C=O) stretching in the range of 1715 ± 100 cm^−1^ appeared for mPEG-functionalized silica particles due to presence carbonyl groups in the mPEG–silane structure (Figure 1). Note that the carbonyl moiety seen in the mPEG structure is not the part of the repetitive unit, however, is present in the structure due to the coupling chemistry used to prepare mPEG-silane. It was also anticipated that the characteristic broad peak in the range of 3000–3600 cm^−1^ for mPEG-grafted silica would appear due to O−H stretching [34]. The residual moisture upon grafting and washing steps of mPEG-grafted silica particles with water and ethanol caused the broad −OH peak to appear in the aforementioned region. The presence of the residual moisture content is supported by the very small mass loss seen in the initial phase of the thermogravimetric (TGA) profile where the silica particles were heated to 120 °C and remained for ~3 min (Figure 2). Although an N−H stretching peak is expected in 3000–3600 cm^−1^ region, due to the strong −OH peak, the signal associated with the N−H stretching is likely masked. An −NH group is also present in the structure of mPEG silane due to coupling chemistry used. Moreover, the peak around 2800 ± 100 cm^−1^ appeared is attributed to −CH_2_ stretching vibration from the repetitive units of mPEG grafted on silica particles [33,35]. The small peaks at ~1462 and 1347 cm^−1^ are attributed to −CH_2_ bending and wagging vibration, respectively [33,35]. In summary, identification of characteristic peaks of carbonyl (C=O), N−H, and −CH_2_ suggest that successful coupling of mPEG–silane to silica particles was achieved.

#### 2.1.2. Thermogravimetric (TGA) Analysis of Bare and mPEG-Grafted Silica Particles

Figure 2 shows the percent mass loss of bare and mPEG-grafted silica particles. mPEG-2000 decomposition starts at ~400 °C, whereas mPEG-5000 started to decompose at ~300 °C, which compares to previous research showing a decomposition temperature of 392 °C for mPEG-2000-grafted silica [36]. In order to quantify the mass loss due to mPEG decomposition, the mass loss of bare silica was used as a baseline and any additional mass loss observed in mPEG-grafted silica particles was associated with the decomposition of mPEG. The percent weight loss difference between bare silica and mPEG-2000 was 2.1%, whereas mPEG-5000 had 5.0% additional mass loss compared to bare silica (Figure 2). The additional mass loss observed for mPEG-grafted silica particles suggests that both mPEG-2000 and mPEG-5000 were successfully grafted onto the silica surface. The additional mass loss of 5.0% and 2.1% in mPEG-5000 and mPEG-2000, respectively, compared to bare silica correspond to 0.01 mmol mPEG/g silica for both mPEG-5000 and mPEG-2000. This suggests that grafting densities of mPEG-5000 and -2000 are similar and any differences observed in polyphenol adsorption capacity is associated with the chain length of PEG.

#### 2.1.3. Elemental Analysis of Bare and mPEG-Grafted Silica Particles

Elemental analysis of mPEG-grafted and bare silica particles verified the surface modification. Table 1 shows the C, H, and N composition of bare and mPEG-grafted silica particles. Surface modification with mPEG resulted in higher C, H, and N weight percentages in comparison to bare silica particles where mPEG-5000 showed ~2-fold higher wt % of C and H in comparison to mPEG-2000. This is also in good agreement with TGA analysis showing ~2.5-fold difference in % weight loss between mPEG-2000 and -5000. Increase in the percentage of elemental C, H, and N of mPEG-grafted silica particles in comparison to bare silica is attributed to the successful surface functionalization of silica particles with mPEG ligands. It is worthy to note that bare silica shows ~1% C, ~0.5% H, and ~0.07% N contents, where it is believed that the organic contamination associated with the sample handling during the measurements likely resulted in detection of carbon, hydrogen, and very limited nitrogen content. Similar organic contents of bare silica were reported elsewhere [37,38].

### 2.2. Adsorption of Polyphenols Extracted from Grape Pomace onto mPEG-Grafted Silica Particles

**Figure 3:**
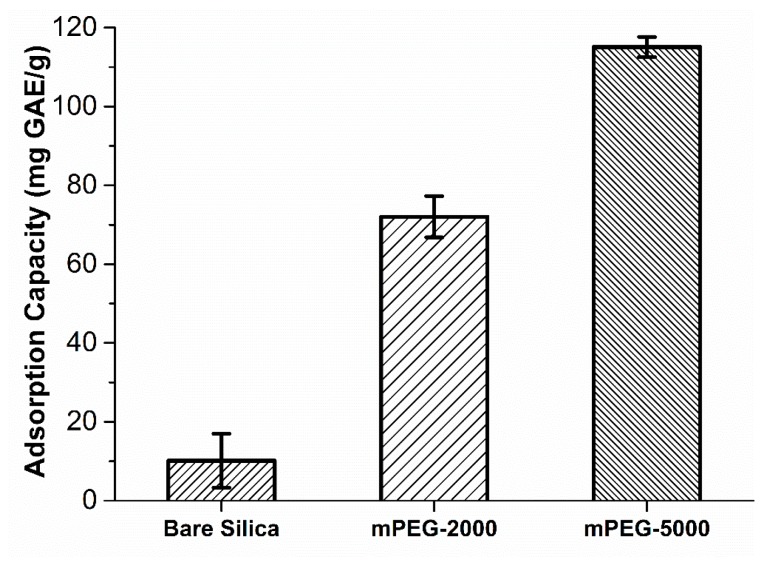
Adsorption capacities of bare, mPEG-2000-, and mPEG-5000-grafted silica particles for total polyphenols as determined by Folin–Ciocalteu method. Adsorption experiments were performed with 25 mg of adsorbents. A statistically significant difference between all the capacity values was observed (*p* < 0.05). Error bars indicate a standard deviation observed for duplicates. GAE: Gallic acid equivalents.

Figure 3 shows the adsorption capacities of the adsorbents used in this study. Bare silica showed only 10.1 mg GAE (gallic acid equivalents)/g adsorption capacity whereas surface grafting with mPEG-2000 and -5000 increased the adsorption capacity by 7.1- and 11.4-fold compared to bare silica, respectively. Higher adsorption capacities observed in mPEG-grafted silica particles compared to bare silica can be attributed to the more polyphenol binding sites. Grafting bare silica with mPEG introduces a high number of ether sites which presumably facilitates hydrogen bonding between polyphenols and mPEG ligands. A similar hydrogen bonding phenomenon can be seen between bare silica and polyphenols. Bare silica surface carries silanol groups which can potentially be involved in hydrogen bonding [39]; however, it is likely that the number of these sites are limited. Therefore, bare silica yields poor polyphenols adsorption capacity. This is in good agreement with other studies showing that bare silica surfaces showed very low quercetin adsorption capacity compared to titania-modified silica particles [40,41]. mPEG-5000-grafted silica particles showed 1.6-fold higher polyphenol adsorption capacity compared to mPEG-2000. Although the conformation of mPEG on the silica surface is not known, it is likely that mPEG-5000 exposes more hydrogen bonding sites compared to mPEG-2000 due to the presence of higher number of repetitive PEG units. Exposing more ether sites would likely increase the probability and affinity of polyphenols to bind the PEG chains resulting in higher adsorption capacities. These results are in good agreement with literature showing that increasing the chain length of PEG from 6 to 43 repetitive units resulted in strong bonding to biomolecules available on cell surfaces, presumably through hydrogen bonding [26]. This unique ability of PEG provides surface tunable properties which regulate the polyphenol adsorption capacity with mPEG-grafted silica particles. In addition to the tunable properties, mPEG-grafted silica particles demonstrated higher adsorption capacities in comparison to traditionally used food-grade polymeric resins. Soto et al. evaluated 13 different food grade resins such as Amberlites, Diaions, and SepaBeads with various structures and reported ~1.5–2.6 mg GAE/g adsorption capacities for polyphenols recovered from winery waste [42]. It is important to note that the surface areas of these resins range from 300 to 1200 m^2^/g. mPEG-2000- and mPEG-5000-grafted silica particles used in our study have less surface area (Table 1) compared to the resins used in Soto et al. [42] while demonstrating ~28–48- and ~44–77-fold higher total polyphenol adsorption capacities, respectively. These results confirm the high potential for mPEG-grafted silica particles to be used in the recovery of polyphenolic compounds present in the winery waste. Since mPEG-5000-grafted silica particles showed the highest polyphenol adsorption capacity, desorption experiments were performed using these particles only.

### 2.3. Recovery of Polyphenols from mPEG-Grafted Silica Particles

#### 2.3.1. Screening Solvent Systems Coupled with Reagents That Can Break Hydrogen Bonding for the Recovery of Polyphenols from mPEG-Grafted Silica Particles

##### Effect of Sorbitol and Salt in Aqueous Ethanol on Polyphenol Recovery

**Figure 4:**
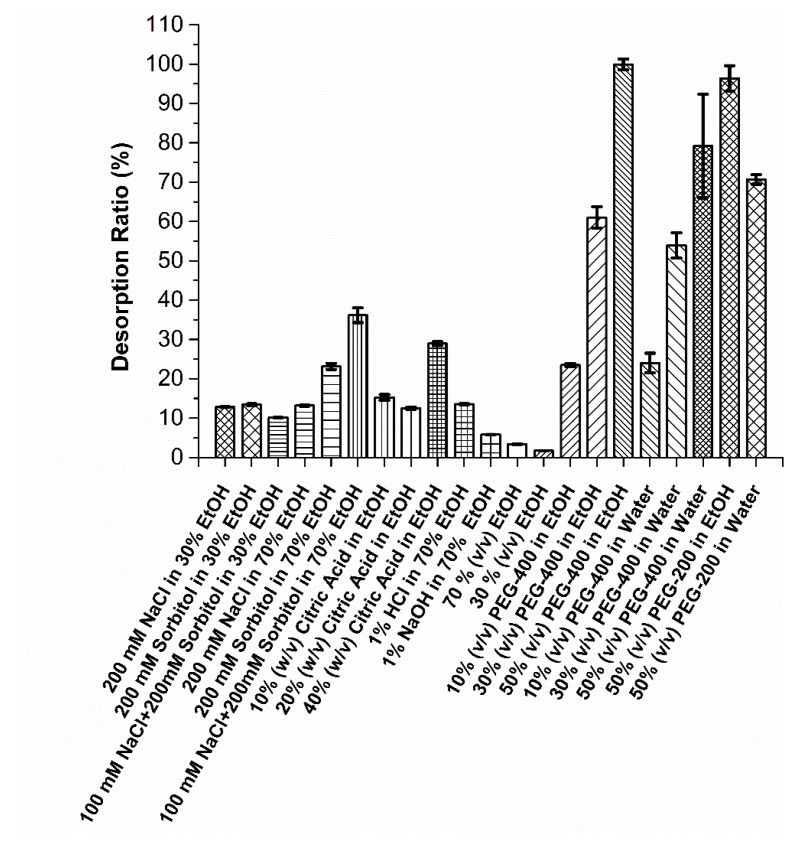
Desorption of total polyphenols from mPEG-5000 grafted silica particles using various solvents. Ethanol is abbreviated as EtOH. A 1 mL desorption solvent was used for recovery of grape pomace polyphenols adsorbed on 25 mg mPEG-5000-grafted silica particles. Error bars indicate a standard deviation observed for duplicates.

Figure 4 shows the desorption ratios of various solvent systems. When sorbitol or NaCl was individually added to 30% aqueous ethanol, the desorption ratios increased by ~8-fold in comparison to 30% aqueous ethanol, yet the desorption ratios were at ~13% level, which is likely considered as a noneffective recovery process for polyphenols. The stronger elution power, seen with the addition of NaCl to the aqueous ethanol, can be partially explained by the salt effect seen in the reverse phase chromatography [43]. When NaCl and sorbitol were added to 70% aqueous ethanol, desorption ratios increased by ~4- and 7-fold in comparison to 70% aqueous ethanol, whereas combined sorbitol/NaCl increased the desorption ratios by ~11-fold, resulting in a desorption ratio of 36.1% (Figure 4). We attempted to use hydrogen bond breaker reagents to release the polyphenols strongly bonded to mPEG-grafted silica particles. Sorbitol is a monosaccharide with six hydrogen donors and acceptors and is commonly used as a biomolecule stabilizer. Sorbitol was previously used to elute large molecule biologics presumably by breaking the hydrogen bonds between the resin and the biomolecule of interest [44]. Moreover, in combination with salts, the elution power was strengthened [44]. In analogy to elution of biologics in hydrogen bonding chromatography [44], sorbitol and NaCl was used in an aqueous ethanol binary solvent system. Although the desorption ratio of 100 mM NaCl + 200 mM sorbitol in 70% aqueous ethanol was promising, further optimization of sorbitol concentration was not performed due to the viscosity constraints.

##### Effect of Acid and Base on Polyphenol Recovery

Acidic and basic conditions were screened to test the ability of these conditions to release the polyphenols from mPEG-grafted silica particles. Both 1% HCl and 1% NaOH in 70% aqueous ethanol showed poor desorption ratios (<14%) (Figure 4), whereas 1% HCl showed ~2-fold higher desorption ratios compared to 1% NaOH, suggesting that acidic conditions are more favorable than basic for the release of polyphenols. However, further optimization was not performed with the strong acids due to the concerns about leaching mPEG ligands from the surface of silica particles [41]. In contrast to strong acids, previous studies using a weak acid—citric acid—showed promising elution performance for the recovery of a model polyphenol—quercetin—from titania-modified silica particles [41]. Citric acid was also supplemented to an extraction solvent used to isolate rutin from *Sophora japonica* [45]. This work, therefore, aimed to increase the desorption ratio by supplementing anhydrous ethanol with varying concentrations of citric acid (Figure 4). Unlike quercetin recovery from titania-modified silica particles using 10 and 20% citric acid in ethanol [41], only ~12 to 15% desorption ratios were observed when polyphenols were eluted from mPEG-grafted silica particles (Figure 4). Increasing the citric acid concentration to 40% increased desorption ratio by ~2-fold in comparison to 10 and 20% citric acid in ethanol with a final desorption ratio of 29%. It was concluded that addition of citric acid was not sufficient to release most of the polyphenols adsorbed onto mPEG-modified silica particles, yet achieved comparable desorption ratios that were observed in 100 mM + 200 mM sorbitol in 70% aqueous ethanol (Figure 4).

#### 2.3.2. Screening PEG Cosolvent Systems for the Recovery of Polyphenols from mPEG-Grafted Silica Particles: Developing PEG–Water and PEG–Ethanol Cosolvent Systems

Due to the limited polyphenol desorption abilities of the solvents discussed above, an alternative solvent system capable of releasing the majority of polyphenols from silica particles was needed. Cosolvent systems are widely used in the pharmaceutical industry to solubilize poorly water-soluble drugs [46]. Propylene glycol (PG), ethanol, glycerin, and PEG-400 are commonly used and the Food and Drug Administration (FDA) approved the cosolvents [47]. A system using PEG-200 or PEG-400 as a cosolvent was developed in this study for the effective release of polyphenols from mPEG-modified silica particles. It is hypothesized that the interactions between polyphenols and mPEG-grafted surfaces are unusually strong [48] and are likely due to the hydrogen bonding network. Therefore, solvents behaving like PEG can possibly break this network effectively. It was intended to create a competition between mPEG on the silica surface and PEG-200 or PEG-400 in the cosolvent and achieve complete recovery of polyphenols from silica particles.

##### Effect of PEG Concentration on Polyphenol Recovery

Figure 4 shows the desorption ratios of PEG–water and PEG–ethanol cosolvent systems with variable PEG concentrations (*v*/*v*). The slight addition of PEG-400 (10%) to ethanol increased the desorption ratio by ~7- and 14-fold in comparison to 70% and 30% aqueous ethanol, respectively (Figure 4). A similar trend was observed when only 10% PEG-400 was added to water, where an increase in the desorption ratio of ~7- and 14-fold in comparison to 70% and 30% aqueous ethanol, was determined, respectively. When the PEG-400 concentration was increased from 10% to 30 and 50% (*v*/*v*) in ethanol, 3- and 4-fold increases in the desorption ratios compared to the 10% solution were observed, respectively. With that, ~99.9% of the adsorbed polyphenols was recovered using 50% PEG-400–ethanol. These high desorption ratios observed in PEG-400–ethanol cosolvent systems are attributed to the ability of PEG to break the strong hydrogen bond network between mPEG-grafted silica particles and polyphenols. It is likely that adding more PEG introduces competition at the polyphenols’ binding sites and induces the release of more polyphenols. Previous research showed that PEG-400 has remarkable polyphenol solubility in comparison to traditional solvents such as alcohol, castor oil, and water [30]. Therefore, polyphenols released in PEG solvent will be an ideal platform for the downstream use of polyphenols.

##### Effect of Water and Ethanol in PEG Cosolvent Systems for the Recovery of Polyphenols

In order to understand the effect of cosolvents in PEG binary solvent systems on polyphenol recovery, ethanol was replaced with water and the concentration of PEG was varied at 10, 30, and 50% levels corresponding to the same concentrations used in ethanolic PEG cosolvent systems. When ethanol was replaced with water in PEG cosolvent systems, the desorption ratios decreased by 7 and 21% in comparison to PEG-400–ethanol cosolvent systems at 30 and 50% levels, respectively (*p* < 0.05) (Figure 4). This suggests that ethanol is the preferable cosolvent for the recovery of total polyphenols from mPEG-grafted silica particles in comparison to water in PEG cosolvent systems. However, PEG–water cosolvent system still performed well compared to the rest of the solvent systems tested in this study (Figure 4). There was no statistical difference between ethanol and water cosolvent at 10% PEG level (*p* > 0.05). This was likely due to low desorption ratios observed with a low amount of PEG available in the cosolvent and therefore the difference between ethanol and water was likely masked at this level. Interestingly, when water and ethanol were interchanged in PEG cosolvent system, the type of polyphenols released from mPEG-grafted silica particles also varied as confirmed by the analysis of individual polyphenols via high-performance liquid chromatography (HPLC) (Table 2). When water was used as a cosolvent in PEG-400 system, gallic acid, p-coumaric acid, (+)-catechin, malvidin chloride, and isoquercetin were recovered more preferably in comparison to ethanol–PEG-400 cosolvent system (Table 2). Similarly, myricetin was recovered more preferably with water in comparison to ethanol in PEG-200 cosolvent systems (Table 2). In contrast to the individual polyphenols discussed above, only kaempferol was selectively recovered by using ethanol in comparison to water in both PEG-400 and PEG-200 cosolvent systems (Table 2). The different abilities of water and ethanol to release variable polyphenols can be attributed to their degree of solvation or, in order words, their ability to break hydrogen bonds to a different extent between various type of polyphenols and mPEG-grafted silica particles. This solvent swing approach provides unique flexibility on the selection of the types of the polyphenols to be recovered from mPEG-grafted silica particles. Moreover, a sequential desorption with water and ethanol cosolvent systems can be performed for the effective fractionation of the polyphenol of interest.

##### Tunable Recovery of Polyphenols Using Cosolvent Systems with PEG-200 and PEG-400

In contrast to adsorption capacity (Figure 3), altering the MW of PEG in the desorption medium did not affect the desorption ratio because there was no statistical difference between 50% PEG-400 in both water and ethanol and 50% PEG-200 in both water and ethanol (Figure 4) (*p* > 0.05). However, the type and amount of specific polyphenols recovered from the silica particles varied when PEG-400 was replaced with PEG-200 in the desorption medium (Table 2). Table 2 shows the relative peak areas of eluted polyphenols as a function of different solvent systems for individual polyphenolic compounds. When PEG-200 was used in the desorption medium, myricetin and procyanidin-B2 were selectively recovered from silica particles, whereas there was no detectable myricetin and procyanidin-B2 present in PEG-400 cosolvent systems after elution. On the other hand, there was no detectable gallic acid, *p*-coumaric acid, and isoquercetin present in PEG-200 solvent systems (Table 2). A recovery of ~40–50% of gallic, *p*-coumaric, and isoquercetin from grape pomace extract was achieved by the use of a 50% PEG-400–water cosolvent system. In addition, ~82% of malvidin chloride was recovered using the 50% PEG-400–water cosolvent system, whereas only ~28% of malvidin chloride was recovered using 50% PEG-200–water cosolvent system (Table 2). It is worthy to highlight the biological activity of malvidin as it is one of the most abundant polyphenol types isolated with PEG cosolvent systems. Theoretical studies showed that malvidin carries high antioxidant activity [49] and experimental studies demonstrated the ability of malvidin to inhibit the growth of human tumor cells *in vitro* [50]. These higher recovery percentages obtained in PEG-400 cosolvent systems in comparison to PEG-200 cosolvent systems are attributed to the ability of PEG-400 to break hydrogen bonds between these polyphenols and mPEG-grafted silica particles better in comparison to PEG-200. Since the hydrogen bond is directional and the strength of the bond depends on the dipole moment of two interacting moieties [51], the ability of different cosolvents to break these bonds likely varies. Variability in the relative peak areas of polyphenols released from mPEG-grafted particles with various solvents is attributed to the heterogeneity of the structures observed for different polyphenols (Table 2). Interestingly, recovery of delphinidin chloride was not sensitive to the type of the solvents used for the release of the polyphenols from silica particles (Table 2). No detectable quercetin was identified in the desorption medium for all of the solvents tested and, similarly, a very limited amount of (+)-catechin (~0.1–1%) was recovered with PEG-200 and PEG-400 cosolvents system (Table 2). It is worthy to note that there are considerable amounts of (+)-catechin and quercetin present in the grape pomace extract compared to other polyphenols investigated (Table S1). These poor affinities of elution solvents and mPEG-grafted silica particles to quercetin and (+)-catechin suggest that desorption medium and mPEG-grafted silica particles are selective for certain polyphenols.

### 2.4. Antioxidant Activity of the Polyphenols Recovered by the Use of PEG Cosolvent Systems from mPEG-Grafted Silica Particles

**Figure 5:**
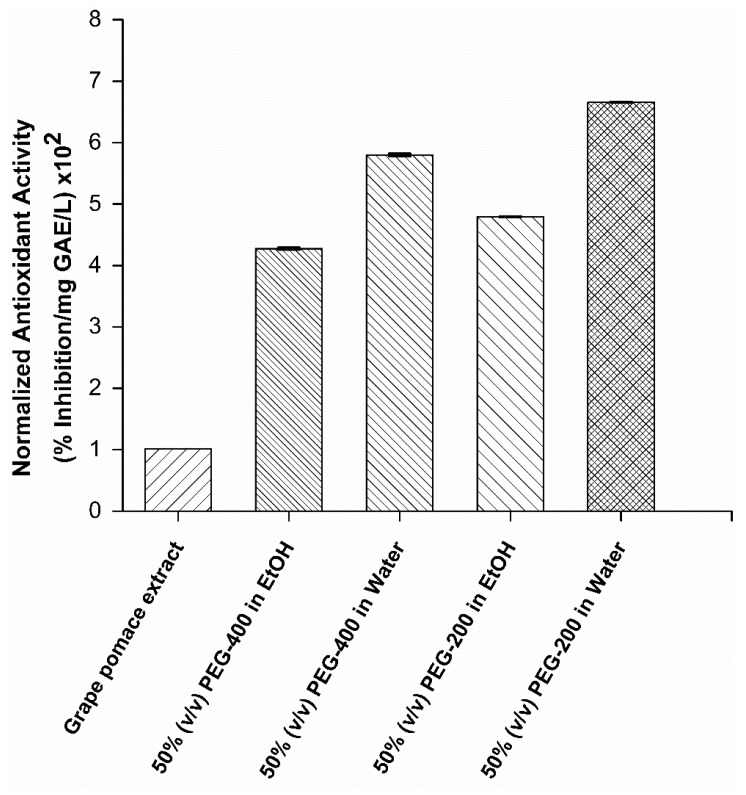
Normalized antioxidant activity of grape pomace extract and polyphenols recovered from mPEG-5000-grafted silica particles using various cosolvent systems. Ethanol is abbreviated as EtOH. A statistical difference was observed for all measurements (*p* < 0.05) Error bars indicate a standard deviation observed for duplicates.

Figure 5 shows the antioxidant activity of grape pomace extract and recovered polyphenol mixture using various desorption solvents. In order to compare the antioxidant activity of grape pomace extract and recovered polyphenols, the antioxidant activity was normalized based on total polyphenol content present in the grape pomace extract and recovered polyphenols, respectively. This allowed us to evaluate the effect of activity observed due to polyphenol composition and not the amount of polyphenols used in the assay. In all cases, recovered polyphenols with mPEG-grafted silica particles showed stronger antioxidant activity in comparison to grape pomace extract, while the degree of antioxidant activity varied with the PEG percentages in the cosolvent and the types of the secondary solvent used (Figure 5). It is generally believed that the antioxidant activity of polyphenols is facilitated through hydroxyl groups available in the structure [44]. Since the hydroxyl groups are likely involved in the hydrogen bonding of polyphenols to mPEG-grafted silica particles, it is attributed that the recovery of polyphenols via hydrogen bonding mechanism would yield better antioxidant activity compared to grape pomace extract. In support of this statement, polyphenols recovered with 50% PEG-200–water showed the highest potency in comparison to polyphenols recovered using three other solvents used in this study (Figure 5). The high potency observed in PEG-200 can be explained by the higher content of both myricetin and procyanidin-B2 in comparison to PEG-400 cosolvent systems (Table 2). Previous antioxidant assays showed that myricetin and procyanidin-B2 have the highest potencies among 20 well-known polyphenols including gallic acid, resveratrol, kaempferol, quercetin, and (+)-catechin [52]. In addition to its antioxidant activity, myricetin shows significant health promoting activities such as anticarcinogenic, antiviral, and antidiabetic activities. An extensive review of the biological effects of myricetin is provided elsewhere [53] and this highlights the importance of the preferable recovery of myricetin using 50% PEG-200–water cosolvent systems. PEG-400–water showed 1.2-fold higher potency in comparison to PEG-400–ethanol (Figure 5). The high potency observed in PEG-400–water is attributed to ~3.5-fold higher gallic acid content in comparison to PEG-400–ethanol (Table 2). Gallic acid was listed as one of the highest potent polyphenols [52] after myricetin and procyanidin-B2. Since PEG-400 cosolvent systems do not contain these polyphenols, it is likely that higher gallic acid content would result in better antioxidant activity seen in the PEG-400–water cosolvent system. Overall, it is likely that enrichment of gallic acid, procyanidin-B2, and myricetin by the use of mPEG-grafted silica particles and PEG cosolvent elution resulted in better antioxidant activity in comparison to grape pomace extract.

## 3. Materials and Methods

Several spectroscopic, gravimetric, and analytical techniques were used for the characterization of the adsorbents and polyphenols. Surface modification of silica particles was assessed by Fourier transform infrared (FTIR), elemental, and thermogravimetric (TGA) analyses. Surface area and pore volumes of silica particles were estimated by N_2_ adsorption using the Brunauer–Emmett–Teller (BET) method. Grape pomace extract and purified polyphenols were characterized with high-performance liquid chromatography (HPLC) and, finally, potency of recovered polyphenols was assessed using an antioxidant activity assay.

### 3.1. Chemicals and Reagents

2,2-Diphenyl-1-picrylhydrazyl (DPPH), Folin–Ciocalteu reagent, formic acid, and sorbitol were purchased from Sigma Aldrich (St. Louis, MO). Spherical silica particles (surface area: 270–300 m^2^/g, particle size: 75–200 μm, total pore volume: 0.7–0.9 cm^3^/g, as specified by the manufacturer) were also obtained from Sigma Aldrich (St. Louis, MO, USA). Sodium carbonate, sodium chloride, HPLC-grade acetonitrile and methanol, hydrochloric acid, sulfuric acid, sodium hydroxide, and hydrogen peroxide were purchased from Fischer Scientific Co. (Pittsburgh, PA, USA). Methoxy polyethylene glycol Silane-2000 and -5000 (mPEG–silane) were purchased from Laysan Bio (Arab, AL). Analytical reference materials were obtained as follows: Gallic acid, (+)-catechin, delphinidin chloride, p-coumaric acid, quercetin, and kaempferol were supplied from Millipore Sigma (St. Louis, MO, USA); procyanidin B2, isoquercetin, malvidin chloride, and myricetin were purchased from Cayman Chemical (Ann Arbor, MI, USA). Water was purified using a Barnstead E-Pure system (ThermoFisher Scientific, Waltham, MA, USA) with a resistivity of 18 MΩ-cm unless otherwise specified.

### 3.2. Preparation of Grape Pomace Samples

Grape pomace (grape skin, seeds, and stem) from red winemaking using merlot grapes harvested in 2017 was kindly provided by Chateau Ste. Michelle Winery (Woodinville, WA). Upon receipt of grape pomace, the samples were frozen at −20 °C and lyophilized. Then, the dried grape pomace samples were ground to powder (>40 mesh) and stored in plastic bags at −20 °C until used for further experiments.

### 3.3. Preparation of Grape Pomace Extracts

An aqueous ethanol solvent extraction was performed to isolate the polyphenolic compounds from grape pomace. The polyphenolic compounds were extracted at a solid to liquid ratio of 1:10 (*w*/*v*) using 70:30 (*v*/*v*) ethanol: water as the extraction solvent. Following 15 min of sonication treatment, the extraction was performed at 30 °C in an orbital shaker in dark. After 24 h extraction, the grape pomace extract was filtered through Whatman 42 ashless filter paper. The resulting extract was stored at −20 °C until used in further experiments.

### 3.4. Preparation of Silica Particles with Functional Groups

Silica particles were selected as the support material for the surface modification. Surface modification of silica particles was performed by using methoxy polyethylene glycol Silane-2000- and -5000 (mPEG), a type of polyethylene glycol, as the functional groups. A cleaning procedure was performed by sonicating the silica particles in water and ethanol, respectively. Silica particles were dried at 105 °C. Then, silica particles were further washed with piranha solution (4:1 (*v*/*v*) H_2_SO_4_: H_2_O_2_) for an hour at 90 °C to remove the organic impurities. *Caution: piranha solution should be handled with extreme care due to its high reactivity.* Piranha-solution-treated silica particles were washed with water followed by ethanol. Then silica particles were dried in a stream of nitrogen and stored in a nitrogen environment until further usage. The surface modification of silica particles was performed via a silanization process [54,55]. During the silanization reaction, ethoxysilane moieties available in the mPEG silane (Figure 1) are detached with the coupling reaction and Si-O-Si bonds between Si atoms of mPEG and silica surface are formed [54,56]. The structure of mPEG silane contains −NH and −CO moieties as indicated by the manufacturer (Figure 1). These two moieties and the repetitive ethylene glycol units will remain on the surface of the silica particles upon silanization. It is worthy to note that the number of −NH and −CO moieties compared to repetitive ethylene glycol units are significantly low. The silanization of 1.0 g of dried silica particles was performed in a 3 mM mPEG–silane solution with a working volume of 10 mL toluene [54]. The silanization reaction was performed at room temperature for 18 h under a nitrogen environment. After silanization, the particles were washed with toluene, ethanol, and water twice for each washing steps. The silica particles were then dried in a nitrogen stream and stored in a nitrogen environment until further use.

### 3.5. Characterization of Silica Particles

#### 3.5.1. FTIR Spectroscopy

Surface functionality of bare and mPEG-grafted silica particles was characterized using a Fourier transform infrared spectrometer with an attenuated total reflectance attachment (Shimadzu, Japan). The FTIR spectra were recorded at a resolution of 4 cm^−1^ over 64 scans in the range of 4000–400 cm^−1^.

#### 3.5.2. Elemental Analysis

Elemental analyses of bare and PEG-grafted silica particles were performed using a TruSpec-CHN Micro elemental analyzer (LECO, Saint Joseph, MI, USA) according to the corresponding ASTM procedures (D-5291, E-777, E-778). Briefly, 0.15 g of dried samples were analyzed to determine the total carbon (C), hydrogen (H), and nitrogen (N) content.

#### 3.5.3. TGA

TGA was used to characterize the grafted mPEG groups on the surface of silica particles. A thermogravimetric analyzer, SDTA 851 (Mettler Toledo, Columbus, OH, USA) equipped with STARe data analysis software was used. Approximately 4 mg dried sample was heated under nitrogen flow with a heating ramp of 50 °C/minute from room temperature to 120 °C. The sample was maintained at 120 °C for 3 min to ensure the removal of remaining water/solvent in the sample prior to a new 100 °C/minute heating ramp to 950 °C, where the sample was kept for 5 min. The percent mass loss between 120 °C to 950 °C was calculated for both bare and mPEG-grafted silica particles.

#### 3.5.4. Determination of the Surface Area and Pore Volume of Bare and mPEG-Grafted Silica Particles

The surface area of mPEG-grafted silica particles was determined in order to compare the adsorption capacity of mPEG-grafted silica particles and commonly used polymeric adsorbents without incorporation of the surface area effect. Surface areas and pore volumes of bare and mPEG-grafted silica particles were estimated by nitrogen (N_2_) gas physisorption analysis with Micromeritics TriStar II PLUS Surface Area and Porosity Analyzer (Norcross, GA, USA). All samples were degassed at 40 °C under a vacuum of 0.05–0.1 mbar for 18 h. N_2_ gas adsorption studies were performed at 77 K and in the partial pressure range of p/p^o^ = 0.0001 to 0.99. Surface area and pore volumes were estimated by N_2_ adsorption using the Brunauer–Emmett–Teller (BET) method [57].

### 3.6. Analysis of Total Polyphenols

The total polyphenol contents of grape pomace extracts and purified extracts were determined using the Folin–Ciocalteu colorimetric method [58,59]. Briefly, 20 µL of sample was mixed with 1.58 mL water and 100 µL of Folin–Ciocalteu reagent. After 8 min, 300 µL of 20% sodium carbonate solution was added. The mixture was vortexed for 10 s and then incubated at 40 °C for 30 min. The absorbance was measured at 765 nm using ultraviolet-visible spectrophotometer (Shimadzu, Japan). Total polyphenolic contents of samples were expressed as gallic acid equivalents (GAE) in mg L^−1^ of bulk solution. It is important to note that individual polyphenols can show different gallic acid equivalency [60,61]. Although two different mixtures of polyphenols result in the same GAE, the distribution of the individual polyphenols in a given mixture can significantly differ from the other mixture. Therefore, the composition of the grape pomace extract and recovered polyphenols were further characterized via HPLC.

### 3.7. Batch Adsorption and Desorption of Polyphenols

It is well known that surface functional groups regulate the ability of the adsorbents to capture the molecule of interest. Here, the ability of mPEG grafting to bind polyphenols from grape pomace extract was tested. Bare silica particles were used as a control for evaluating the effect of mPEG grafting on polyphenol recovery from grape pomace extract. The batch adsorption experiments were performed in 2 mL centrifuge tubes loaded with 25 mg dry adsorbents. Prior to adsorption experiments, the bare and mPEG silica particles were prewetted with 1 mL of extraction solvent (70:30 (*v*/*v*) ethanol: water) for 2 h. The particles were centrifuged for 15 min at 15,000× *g* and the supernatant was removed. Then, 1 mL of grape pomace extract (9.1 ± 0.3 mg/mL) was loaded to the prewetted particles. The tubes were shaken in an orbital shaker at 125 rpm for 24 h at 25 °C in dark. After the attainment of adsorption equilibrium, the supernatant was collected and analyzed for total phenolic content. The total phenolic contents of the initial solution and the solution at the equilibrium were measured. The adsorption capacities of the particles were calculated from the difference between the concentrations of initial and final solutions by using Equation (1):

(1) $$Q_{e}=\frac{\left(C_{0}-C_{e}\right)\times V_{0}}{m}$$

where *Q_e_* is equilibrium adsorption capacity (mg/g), *C_e_* is the equilibrium concentration of solute in bulk solution (mg/L), *C_0_* is the initial concentration of solute in bulk solution (mg/L), *V* is the volume of the initial sample solution, and *m* is the amount of dry adsorbent (g).

After acquiring the samples for total phenol analysis, the silica particles were spun and extracts were decanted. Polyphenol-loaded silica particles were washed with 1 mL of extraction solvent (70:30 (*v*/*v*) ethanol: water). After vortex mixing, the particles were centrifuged for 5 min at 15,000× *g* and the supernatant was discarded. Following this wash step, the particles were resuspended in 1 mL of different elution solvents (200 mM NaCl in 30% ethanol, 200 mM sorbitol in 30% ethanol, 100 mM NaCl and 200 mM sorbitol in 30% ethanol, 200 mM NaCl in 70% ethanol, 200 mM sorbitol in 70% ethanol, 100 mM NaCl and 200 mM sorbitol in 70% ethanol, 10–40% (*w*/*v*) citric acid in ethanol, 1% HCl in 70% ethanol, 1% NaOH in 70% ethanol, 70% ethanol, 30% ethanol, 10–50% (*v*/*v*) PEG-400 in ethanol, 10–50% (*v*/*v*) PEG-400 in water, 50% (*v*/*v*) PEG-200 in ethanol, 50% (*v*/*v*) PEG-200 in water) to recover the polyphenols from silica particles. The tubes were shaken in dark at 125 rpm in an orbital shaker. After 24 h, an aliquot of supernatant was collected and analyzed for the total and individual phenolic content. The desorption ratio of the silica particles was estimated using Equation (2):

(2) $$D=\frac{\left(C_{d}V_{d}\right)}{\left(C_{0}-C_{e}\right)V_{0}}\times 100$$

where *D* is percent desorption ratio, *C_d_* is the concentration of solute in desorption solution (mg L^−1^), *V_d_* is the volume of desorption solution (mL), and *C_0_* and *V_0_* are the same as described in Equation (1) [62].

### 3.8. Developing the Green Solvents for the Recovery of Adsorbed Polyphenols onto the mPEG-Modified Silica Particles

Since there is strong interaction expected between mPEG ligands and polyphenols, a robust process for the complete recovery of polyphenols is needed. The release of total polyphenols can be facilitated with an elution step. Choosing the right elution solvent for the recovery process is extremely important due to the strict regulations imposed for food and pharmaceutical industry. Therefore, a “green” solvent approach has to be implemented for the complete recovery of polyphenols from silica particles. Twenty-one different solvent systems were tested in this study aiming to achieve a complete recovery. Moreover, tunable cosolvent systems were designed to facilitate preferable recovery of certain types of polyphenols from mPEG-grafted silica particles.

### 3.9. Antioxidant Activity

Antioxidant activities of the polyphenols in grape pomace extract and the polyphenols recovered from mPEG-5000-grafted silica particles were tested using a 2, 2-diphenyl-1-picrylhydrazyl (DPPH) free radical assay [63,64]. Briefly, the activity of each sample was determined by mixing 20 µL sample with 980 µL 6.1 × 10^−5^ M DPPH• methanol solution in 2 mL cuvettes. The cuvettes were covered and incubated in the dark for 16 min. The absorbance at 517 nm was measured against a blank by using ultraviolet-visible spectrophotometer (Shimadzu, Japan). Since the total polyphenol contents of each sample vary, the results were normalized to the total polyphenolic content of each sample and expressed as normalized antioxidant activity (inhibition percentage (%)/(mg GAE/L)) in Equation (3).

(3) $$Normalized\;Antioxidant\;Activity=\frac{\left(\frac{Absorbance_{t=0\;\min}-Absorbance_{t=16\;\min}}{Absorbance_{t=0\;\min}}\right)}{Total\;polyphenol\;content\;(mg\;GAE/L)}\times 100$$

### 3.10. Analysis of Individual Polyphenols via High-Performance Liquid Chromatography (HPLC)

HPLC analysis of individual polyphenols was performed using an Agilent 1200 series HPLC (Agilent Technologies, Santa Clara, CA, USA) equipped with ChemStation software, a degasser, a quaternary gradient pump, an autosampler, a column oven, and a multiwavelength detector. Separation of polyphenols was achieved using a Poroshell 120 Stable bound SB–C18 column (150 × 4.6 mm, 2.7 µm particle size Agilent Technologies, Santa Clara, CA, USA), with a Poroshell 120-C18 guard column (5 × 4.6 mm, 2.7 µm particle size Agilent Technologies, Santa Clara, CA, USA), operated at 30 °C. The mobile phase consisted of 0.1% formic acid in water (Solvent A) and 0.1% formic acid in acetonitrile (Solvent B). The multiwavelength detector was set to 280 nm. Then, 1 µL of each sample was directly injected after filtering through a 0.2-µm PTFE (Polytetrafluoroethylene) syringe filter. The flow rate was set at 0.3 mL/min. The gradient program started at 0% B and increased to 15% B over the course of 5.25 min and held at 15% B for 5.4 min. The percentage of mobile phase B was then increased to 40% over 26.85 min and kept at 40% B for 1.5 min. The mobile phase was ramped to 100% B in 1.5 min and held at 100% B for 3 min. Then, the gradient program was set to its initial conditions of 0% B within 1.5 min and followed by re-equilibration of the column for 10 min. Individual polyphenolic compounds were identified by comparing the retention times of analytical reference materials. Concentrations of the individual phenolic compounds were quantified using external calibration curves generated for the analytical standards.

### 3.11. Statistical Analysis

All of the experiments were performed in duplicates and data were reported as mean values ± standard deviation. A statistical test was performed to determine the statistical difference between samples investigated using one-way analysis of variance (ANOVA) followed by a pairwise comparison using Tukey test. The mean values were considered to be significant when *p* < 0.05. The mean of the coefficient of variation (CV) of all measurements in this study was 5.3% indicating that duplicate measurements are sufficient to describe the population of the measurements.

## 4. Conclusions

Functionalized silica particles are promising adsorbents for the recovery of polyphenolic compounds from grape pomace. Modifying bare silica surface with mPEG ligands provided a dramatic increase in the adsorption capacity whereas altering the MW of mPEG-grafted on silica surface provided tunability in the adsorption capacity. A complete recovery of polyphenols from mPEG-grafted silica particles was achieved by utilizing PEG–ethanol or PEG–water cosolvent systems. Recovered polyphenols showed up to ~12-fold potency in comparison to grape pomace extract as determined by DPPH antioxidant activity. Alternating the cosolvent from ethanol to water in PEG cosolvent systems provided preferable isolation of certain type of polyphenols adsorbed onto mPEG-grafted silica particles. This study demonstrated the suitability of the use of mPEG-grafted adsorbents and PEG cosolvents for the large-scale recovery of polyphenols from grape pomace extract.

## Figures and Tables

**Table 1:** Elemental composition and physical characteristics of bare and mPEG-grafted silica particles.

	C (wt %)	H (wt %)	N (wt %)	Surface Area (m^2^/g)	Total Pore Volume (cm^3^/g)
**Bare Silica**	1.02 ± 0.01	0.50 ± 0.01	0.07 ± 0.05	270.74 ± 1.45	0.869
**mPEG-2000**	2.28 ± 0.02	0.62 ± 0.00	0.08 ± 0.01	248.29 ± 1.35	0.806
**mPEG-5000**	5.02 ± 0.04	1.24 ± 0.02	0.11 ± 0.00	234.83 ± 1.35	0.768

**Table 2:** Relative peak areas of eluted polyphenols with various solvent systems as determined by high-performance liquid chromatography (HPLC).

Elution Solvent	(%) Recovered Individual Polyphenols ^a^									
	GA ^1^	CA ^2^	PB2 ^3^	DC ^4^	pCA ^5^	IC ^6^	MC ^7^	MY ^8^	QU ^9^	KA ^10^
**50% (*v*/*v*) PEG-400** **in Ethanol**	13.8 ± 0.5	0.2 ± 0.0	ND ^b^	8.8 ± 0.7	10.6 ± 1.5	24.4 ± 1.4	53.7 ± 0.7	ND	ND	52.6 ± 4.7
**50% (*v*/*v*) PEG-400** **in Water**	48.7 ± 1.3	0.4 ± 0.0	ND	9.9 ± 1.0	50.2 ± 1.9	43.5 ± 2.4	82.0 ± 0.9	ND	ND	29.4 ± 0.7
**50% (*v*/*v*) PEG-200** **in Ethanol**	ND	0.1 ± 0.0	7.3 ± 0.3	8.3 ± 0.6	ND	ND	ND	30.39 ± 3.3	ND	52.6 ± 1.4
**50% (*v*/*v*) PEG-200** **in Water**	ND	1.0 ± 0.1	5.7 ± 0.2	9.2 ± 0.6	ND	ND	27.6 ± 0.3	49.3 ± 3.1	ND	14.4 ± 0.5

^a^ (Peak area in the elution/Peak area in the grape pomace extract) × 100, ND: Not detected, ^1^ GA: Gallic acid, ^2^ CA: (+)-Catechin, ^3^ PB2: Procyanidin-B2, ^4^ DC: Delphinidin chloride, ^5^ pCA: p-coumaric acid, ^6^ IC: Isoquercetin, ^7^ MC: Malvidin chloride, ^8^ MY: Myricetin, ^9^ QU: Quercetin, ^10^ KA: Kaempferol. Data are represented in mean ± standard deviation of duplicates.

## Supplementary Materials

The following are available online, Table S1: Individual phenolic compounds of grape pomace extract.
