# Supplementary material for: Recovery of Polyphenols from Grape Pomace Using Polyethylene Glycol (PEG)-Grafted Silica Particles and PEG-Assisted Cosolvent Elution
Source: Molecules. 2019 Jun 12;24(12):2199. doi: 10.3390/molecules24122199 (PMC6630576; doi:10.3390/molecules24122199)
Supplement: Supplementary file 1 [file molecules-24-02199-s001.pdf]

Supporting Information

Recovery of polyphenols from grape pomace using polyethylene glycol (PEG)-grafted silica particles and PEG assisted co-solvent elution

*Ayca Seker *[1], Baran Arslan [2], and Shulin Chen [1]*

[1]Department of Biological Systems Engineering, Washington State University, Pullman, WA, 99164-6120, United States

[2]Gene and Linda Voiland School of Chemical Engineering and Bioengineering, Washington State University, Pullman, WA, 99164-6515, United States

Table S1. Individual phenolic compounds of grape pomace extract

	Individual Polyphenols (mg L^{-1})									
	GA[1]	CA[2]	PB2[3]	DC[4]	pCA[5]	IC[6]	MC[7]	MY[8]	QU[9]	KA[10]
Grape pomace extract	70.2 ±0.6	983.2 ±37.7	288.8 ±2.5	486.7 ± 31.2	47.9 ±5.4	487.3 ±0.9	345.6 ±3.1	10.4± 0.5	275.3 ±0.4	259.4 ±4.7

[1]GA:Gallic Acid, [2]CA:(+)-Catechin, [3]PB2:Procyanidin-B2, [4]DC:Delphinidin Chloride, [5]pCA:P-coumaric acid, [6]IC:Isoquercetin, [7]MC: Malvidin Chloride, [8]Myricetin: MY, [9]Quercetin: QU, [10]Kaempferol: KA. Data are represented in mean ± standard deviation of duplicates.
